# Surface Morphology of Amygdala Is Associated with Trait Anxiety

**DOI:** 10.1371/journal.pone.0047817

**Published:** 2012-10-24

**Authors:** Shuyu Li, Yanan Wang, Pengfei Xu, Fang Pu, Deyu Li, Yubo Fan, Gaolang Gong, Yuejia Luo

**Affiliations:** 1 State Key Laboratory of Software Development Environment, School of Biological Science and Medical Engineering, Beihang University, Beijing, China; 2 Key Laboratory for Biomechanics and Mechanobiology of Ministry of Education, School of Biological Science and Medical Engineering, Beihang University, Beijing, China; 3 State Key Laboratory of Cognitive Neuroscience and Learning, Beijing Normal University, Beijing, China; Institute of Psychology, Chinese Academy of Sciences, China

## Abstract

Previous neuroimaging studies have suggested a role of amygdala in trait anxiety level, in which amygdala was typically treated as a whole. To date, it remains unknown whether the morphology of specific subregions of amygdala are associated with trait anxiety. Here, we employed a shape analysis approach to locate the association between its morphology and trait anxiety on the surface of amygdala. 24 healthy young participants were included. The boundary of amygdala for each subject was first manually outlined using high-resolution magnetic resonance (MR) image, followed by 3D surface reconstruction and parameterization using spherical harmonic description. Two point-wise metrics, direct displacement between the individual surface and atlas surface and its normal projection, were used to quantify the surface morphology of amygdala. Statistical analysis revealed significant correlations between the two surface metrics and trait anxiety levels, which were located around the lateral and central nucleus of right amygdala. Our results provided localized information for the association between amygdala and trait anxiety, and suggested a central role of the lateral and central nucleus of right amygdala on trait anxiety.

## Introduction

It has been well-known that amygdala performs a primary role in emotional processing, such as fear [1], threatening situation [2], [3], and stressful situation [4], [5]. And previous studies have suggested a strong association between structural/functional characteristics of amygdala and trait anxiety, which is the potential, or tendency to undergo uncomfortable experience occurring when a person feels threatened by a situation [6], [7]. For example, using functional magnetic resonance imaging (fMRI), Stein and colleagues found increased activation of bilateral amygdala during emotional processing in anxiety-prone subjects [8]. Several resting-state fMRI studies have reported abnormalities of amygdala-related connectivity in social anxiety disorders, such as dysfunctions of the fronto-amygdala inhibition [9], decreased connectivity between amygdala and visual cortices [10], and increased connectivity between left amygdala and right median prefrontal cortex [11]. Using histochemical techniques, Mora and colleagues revealed that amygdaloid dopamine D2-like receptors that were associated with the modulation of anxiety responses had a topographically differentiated distribution within amygdala of rats, mainly in the central amygdaloid nucleus [12]. In addition, decreased volume of entire left amygdala was observed in individuals with higher state/trait anxiety measures [13].

In contrast to the traditional volumetric analysis, surface morphology analysis employs various shape modeling approaches to represent the surface of various anatomical structures, allowing for locating regional deformations on the surface [14]. A large body of research has explored the surface morphology of brain structures, such as the central sulcus [15], hippocampus [16]–[18], and amygdala [19]–[23]. The key advantage of surface morphology analysis was the ability of detecting subtle differences (i.e., that could not be captured by volumetric measures) on the surface of anatomical objects between subjects. For instance, Posener and colleagues found no differences in hippocampal volume between depressed patients and controls, but highly significant group differences in hippocampal subregional shape using surface morphology analysis [24]. Similarly, Tamburo and colleagues observed significant shape differences on the surface of amygdala, rather than the volume, between late-life depression patients and normal subjects [25].

Using surface morphology analysis, a few studies have examined changes of surface morphology of amygdala with affective disorders. For instance, significant surface contraction around basolateral nucleus of bilateral amygdala has been reported in late-life depression subjects [25]. In addition, Peterson and colleagues have found increased area in the dorsal and ventral surfaces of amygdala in Tourette syndrome group [26]. However, it remains unknown whether and how the surface morphology of amygdala is associated with trait anxiety level across normal subjects.

Here, we investigated the association between 3D surface morphology of amygdala and trait anxiety in 24 healthy young participants. Given the central role of amygdala in emotional processing, we expected to observe location-specific correlations between surface morphological measures and trait anxiety. Specifically, we used quantitative trait measure in State-Trait Anxiety Inventory (STAI-T) [27] to quantify the level of trait anxiety for participants. The anatomical boundary of amygdala for each subject was manually outlined by using high-resolution structural magnetic resonance (MR) image, followed by 3D surface reconstruction and parameterization using spherical harmonic description. A general linear model was applied to detect significant correlations between surface metrics and STAI-T scores.

## Materials and Methods

### Participants

The present study included data from 25 young adults (males, 13; females, 12; age, 17–24 years). All subjects were recruited from local community and campus. There is no history of neurological and psychiatric disorders for all subjects. Our protocol was approved by the Research Ethics Committee of the Beijing Normal University. Informed written consent was obtained from each subject. One image from a male was excluded because of insufficient quality of the structural image (final n = 24).

All participants were asked to finish the STAI-T, and the scores (40±7.56) were used to quantify the level of trait anxiety. In addition, the self-rating depression scale (SDS) [28] was tested for each subject, as well.

### Image Acquisition and Preprocessing

All scans were performed on the same 3.0 T Siemens Tim Trio MRI scanner in the Imaging Center for Brain Research, Beijing Normal University. High-resolution 3D T1-weighted images were sagitally acquired by magnetization prepared rapid gradient echo (MPRAGE) sequence: echo time = 3.44 ms; repletion time = 1900 ms; inversion time = 900 ms; matrix = 256×256; in-plane resolution = 1×1 mm; 176 sagital slices; thickness = 1 mm; and NEX = 1.

All native MR images were first registered into stereotaxic space [29], using a linear transformation [30]. Simultaneously, images were corrected for nonuniformity artifacts using N3 algorithms [31].

### Manual Delineation of Amygdala

Raters were blind to the trait anxiety levels of all subjects. The amygdala boundary in each coronal slice was manually traced using itk-SNAP (http://www.itksnap.org) software, with which raters could check the three orthogonal planes at the same time. The amygdala was delineated according to the protocol that was developed by the Center for Interdisciplinary Brain Sciences Research (CIBSR) at the Stanford University School of Medicine (http://cibsr.stanford.edu) [32].

**Figure 1:**
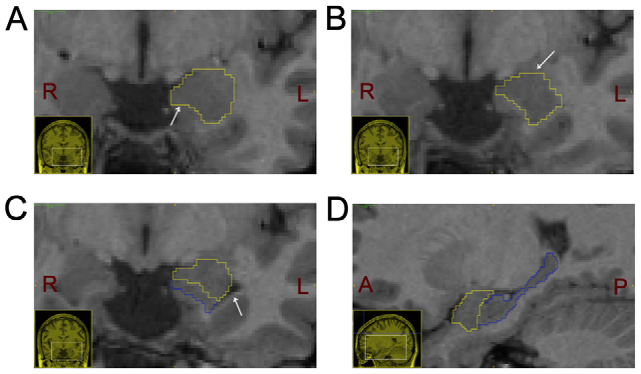
Manual delineation of the amygdala. A) The left amgydala section in the coronal plane. The yellow line represents the contour of amygdala and white arrow indicates its inferior border on coronal plane. Picture in lower left corner is the thumbnail of brain in coronal plane. L and R represent the left and right side, respectively. B) The left amgydala section in the coronal plane. The yellow line represents the contour of the amygdala and white arrow indicates its superior border. C) The left amgydala and hippocampus sections in the coronal plane. The yellow line represents the contour of amygdala and blue line describes the border of hippocampus, the white arrow indicates the temporal horn which begins to enlarge more superiorly along the lateral side of the two structures. D) The left amgydala and hippocampus sections in the sagittal plane. The yellow line represents the contour of amygdala and blue line describes the border of hippocampus. Picture in lower left corner is the thumbnail of the brain in sagittal plane. A and P represent the anterior and posterior side, respectively.

The most anterior slice of amygdala appeared where anterior commisure (AC) was the clearest (thickest, longest, and most continuous). The inferior border was always marked by a white matter tract just under the amygdala (see the arrow in Fig. 1A). As for the medial border, it was formed by either high-intensity white matter or cerebrospinal fluid (CSF). The superior border of the amygdala was marked by a light white matter tract or a CSF boundary (see the arrow in Fig. 1B). The lateral border of the amygdala was marked by the thick, central white matter tract of the temporal lobe. In some cases, the superior lateral border was not clearly defined and we drew an arcing line from the superior border to the white matter boundary on the lateral side of the amygdala [33], [34]. The most challenging part was the segmentation of transition (jumping) slice where both the amygdala and hippocampus were present. On these slices, a band of white matter or CSF could be seen dividing the two structures while the temporal horn began to enlarge more superiorly along the lateral side of the two structures (see the arrows in Fig. 1C). Raters must carefully find out these slices and separate the amygdala and hippocampus apart, at the same time, make the sagittal view as a guide [35] (see Fig. 1D).

To estimate the reliability of the manual outlining, two raters blind to the side of the brain (left or right) traced the boundary of amygdala on five randomly selected brain volumes. Correlation coefficients between the raters for the volumes of the left and right amygdala were 0.90 and 0.91, respectively. After six months, one rater repeated the delineations of amygdala in five randomly selected subjects, and the intra-rater correlation coefficients were 0.96 for left side and 0.93 for the right side.

### Surface Modeling and Registration

We performed the surface reconstruction using SPHARM Modeling and Analysis Toolkit (SPHARM-MAT) (http://www.iupui.edu/shenlab). The spherical harmonic description method was one of the best shape descriptors for amygdala [36].

To ensure that objects had a closed and connected spherical topology, the topology of each manually 3D binary-segmented image was fixed before surface modeling. The resulting images were then parameterized based on spherical mapping. Briefly, a one-to-one bijective mapping was created from the object surface to the unit sphere (with spherical coordinates parameter domain) using Control of Area and Length Distortions (CALD) method, which minimized area distortion cost (ADC) as well as controlled worst average length distortion cost (LDC) [37]. After surface parameterization, each point on object surface had a spherical coordinate representation. Then, each object surface was expanded into a complete set of spherical harmonic basis functions. In this way, the spherical coordinates were represented as the combinations of spherical harmonic basis functions and the coefficients. The object surface can be reconstructed by selecting the coefficients of different degrees. More coefficients results in a more detailed reconstruction [37].

Surface registration was to build the correspondences of the points on the different surface models and facilitate further comparisons across subjects. The procedure mainly included three steps. Firstly, for each object, the first order ellipsoid (FOE) constructed by selecting the first order spherical harmonic coefficients was aligned, and the correspondences across all surfaces were established. Secondly, the resulting surfaces were averaged to generate a template atlas for each side of amygdala. Finally, the surface of left or right amygdala of each subject were further registered to the corresponding template using SPHARM Registration with ICP (SHREC) algorithm, by minimizing the mean square distances between the corresponding points in the object surface and template [37].

### Surface Metrics

**Figure 2:**
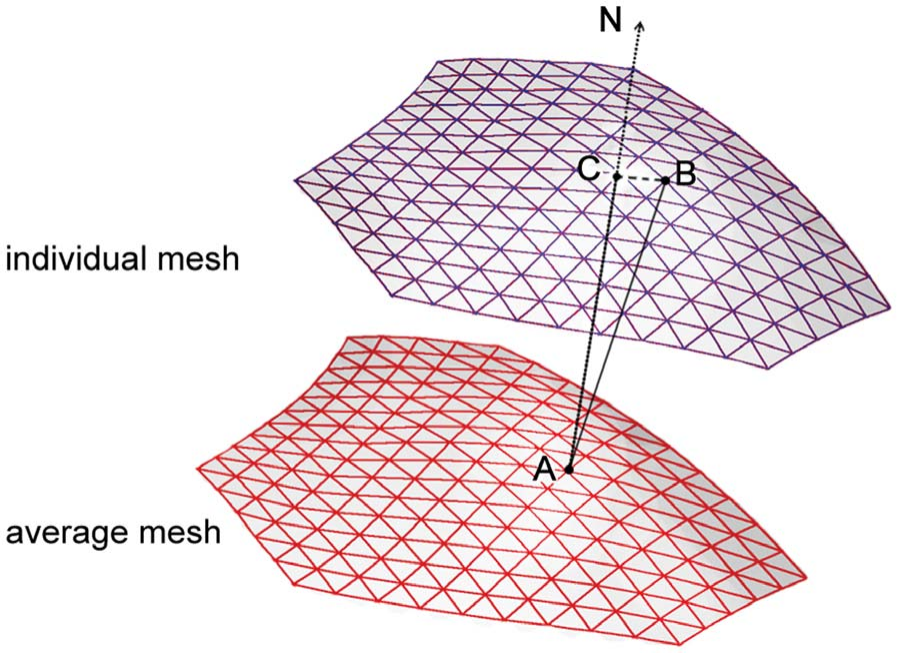
Demonstration of direct displacement and normal displacement. The red surface mesh represents the altas surface constructed from all subjects, and the purple is an individual surface. Point A and B represent two corresponding points on the two surfaces. The black segment between point A and point B represents the direct displacement vector. N is the normal direction of point A on the average surface. The black segment between point A and point C represents the normal displacement (i.e. the projection of the direct displacement).

We adopted two surface metrics that were widely used to quantify the shape difference between individual amygdala and the template. One metric was the direct Euclidean distance between the points in the individual surface and template (i.e. the average surface) [21]. The other metric was defined as the projection of displacement between each point in each subject and the corresponding point in the template to the unit normal of this point in the template surface [38] (see Fig. 2). Here, a positive value of the metrics indicated that the individual regional surface had outward deformation with respect to the template surface and vice versa. Each metric map was blurred using a 5 mm surface-based diffusion smoothing kernel [39].

### Statistical Analysis

To determine whether the surface metrics of amygdala have significant linear correlations with trait anxiety, we performed point-wise general linear models (GLM) with the surface metric as independent variable and STAI-T score as dependent variable. To correct for multiple comparison, random field theory was applied [40]. Specifically, SurfStat (http://www.math.mcgill.ca/keith/surfstat/) was used to implement the statistical analysis and *p*<0.05 (corrected, cluster-level) was considered significant. Given the fact that trait anxiety was mixed with depression [41], we further performed the GLM analysis after controlling for SDS score in the surface clusters that showed significant direct correlations without controlling for SDS.

## Results

### Amygdala Volumetry

The number of voxels in the mask of manually-segmented amygdala was counted as the volume for both left and right amygdala. The volumes of all subjects (n = 24) are 1895±176 mm^3^ for left amygdala, and 1937±159 mm^3^ for right amygdala. There was no significant correlation between the volumes of bilateral amygdala and STAI-T scores (left: *r* = −0.38, *p* = 0.068; right: *r* = 0.19, *p* = 0.385).

### Local Shape Variation

**Figure 3:**
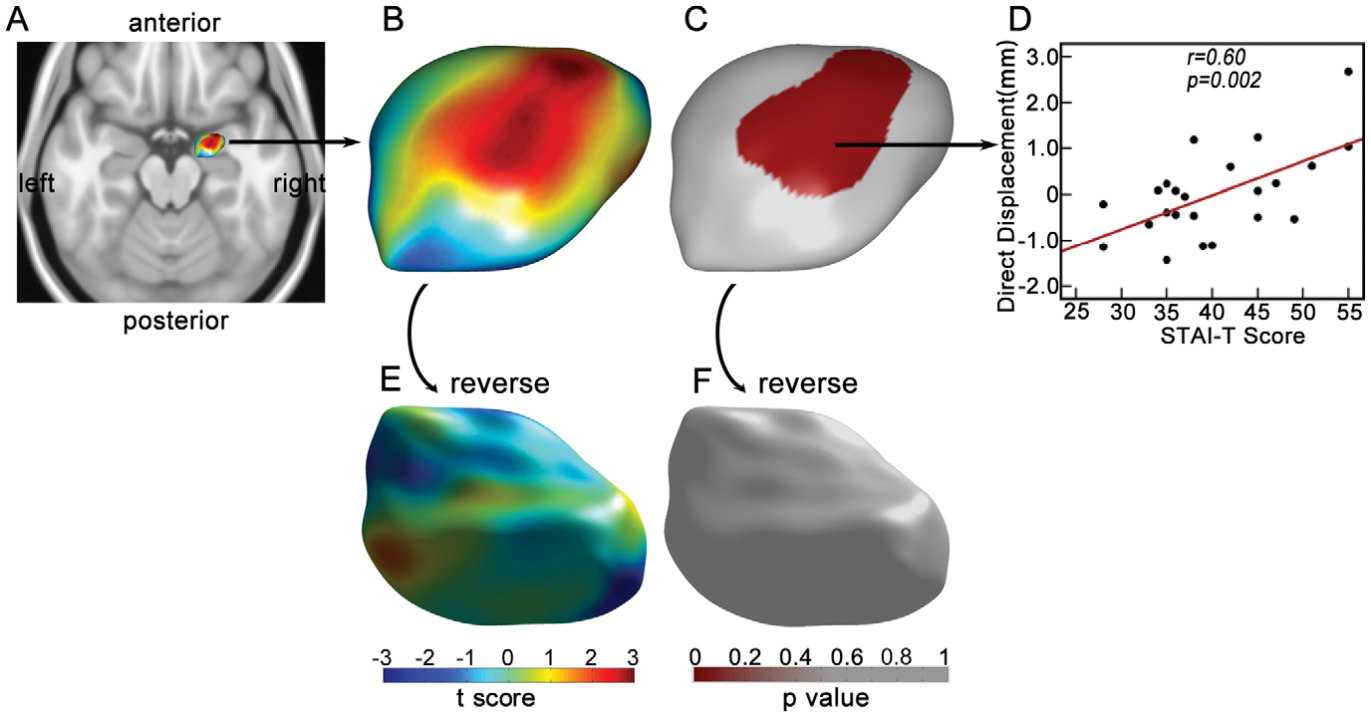
The correlation between direct displacement and trait anxiety. A) The rendering of right amygdala. The figure is shown from direction of superior to inferior. B) Dorsal view of T-statistic map for correlation between direct displacement of right amygdala and STAI-T scores. C) Dorsal view of P-statistic map. Only significant surface clusters (p = 0.037, cluster-level, corrected) are colored. Only one cluster (red) was survived as shown. D) The scatter plot showing averaged direct displacement within the surface cluster versus STAI-T score. The red line is the regression line. E) Ventral view of T-statistic map. F) Ventral view of P-statistic map. Notably, left amygdala was not shown because no significant results were found.

**Figure 4:**
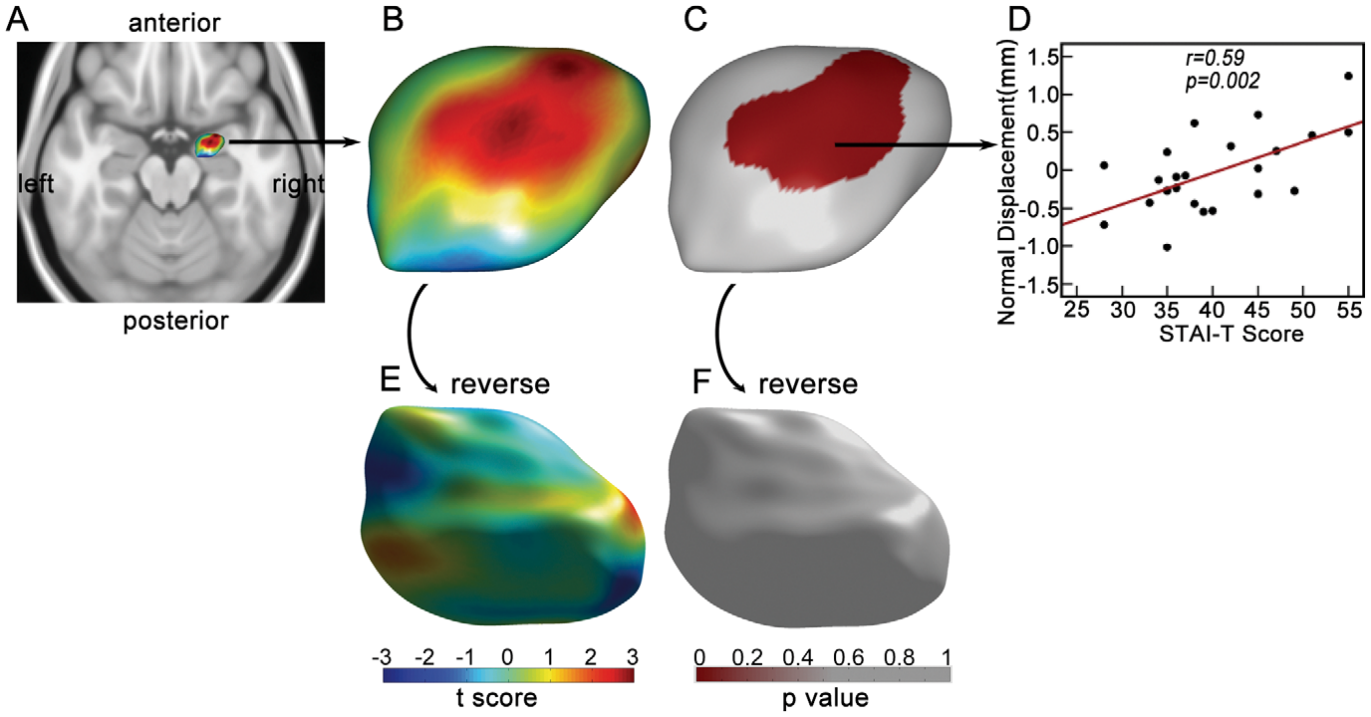
The correlation between normal displacement and trait anxiety. A) The rendering of right amygdala. The figure is shown from direction of superior to inferior. B) Dorsal view of T-statistic map for correlation between normal displacement of right amygdala and STAI-T scores. C) Dorsal view of P-statistic map. Only significant surface clusters (p = 0.043, cluster-level, corrected) are colored. Only one cluster (red) was survived as shown. D) The scatter plot showing averaged normal displacement within the surface cluster versus STAI-T score. The red line is the regression line. E) Ventral view of T-statistic map. F) Ventral view of P-statistic map. Notably, left amygdala was not shown because no significant results were found.

Statistical analysis showed significant positive corrections between direct displacement and STAI-T score in one surface cluster (*p* = 0.037, *cluster-level, corrected*), which is around the lateral and central nucleus of right amygdala. The cluster is displayed in Fig. 3. Consistently, significant positive correlation between normal displacement of amygdala surface and STAI-T score was observed in one cluster with a very similar location (*p* = 0.043, *cluster-level, corrected*), as shown in Fig. 4. There were no significant correlations in left amygdala, using either of the two surface metrics (*p*>0.1, *cluster-level, corrected*).

As expected, significant positive correlation was found between the STAI-T score and SDS score (*p* = 0.008). In order to control for the effect of depression level on morphology of amygdala, we further tested the correlation of surface metrics with STAI-T scores within the significant surface cluster of right amygdala (Fig. 3 and Fig. 4), after controlling for SDS scores. For both surface metrics, the correlation between the averaged surface metrics of the cluster and STAI-T scores remains significant, after controlling for SDS scores (direct displacement, r = 0.54, *p* = 0.008; normal displacement, r = 0.53, *p* = 0.01).

## Discussion

In this study, we investigated the association between surface morphology of amygdala and trait anxiety. Significant positive correlation was observed between surface shape around the lateral and central nucleus of right amygdala and STAI-T score. After controlling for depressive level, we still found significant correlation between surface morphology and trait anxiety in the same location. These results suggested a central role of the lateral and central nucleus of right amygdala in trait anxiety, which furthered our understanding of the neural basis for trait anxiety.

Notably, our results showed that the association of morphology with trait anxiety was confined to the right amygdala. This is compatible with two hypotheses concerning the lateralization of emotion processing [42]: the right hemisphere hypothesis [43] and the valence hypothesis [44]. The right hemisphere hypothesis suggests that all emotional processing is lateralized to the right hemisphere, whereas the valence hypothesis suggests that positive emotion is processed in the left hemisphere, whereas negative emotions are processed in the right hemisphere. Particularly, Bourne and colleagues explored the associations between patterns of lateralization and anxiety, and found that participants with high levels of trait anxiety were more lateralized to right hemisphere for processing facial emotion [42]. More directly, Schienle and colleagues found that trait anxiety was positively correlated with the activation of right amygdala in young healthy volunteers [45]. In addition, generalized anxiety disorder (GAD) patients showed greater activation to fearful faces than to happy faces in right amygdala, while attending to their own subjective fear [46]. In line with these previous findings, our results further supported the notion that right amygdala is in charge of reacting to negative emotion inputs.

The observed positive correlation indicates that the subjects with higher trait anxiety level had more outward deformation in right amygdala. This might implicate outward positioning shifts of right amygdala surface with trait anxiety level. Another possible implication is that more outward deformation represents a larger right amygdala. This is compatible with a recent finding showing larger right amygdala in GAD patients [47]. However, we failed to find significant correlations between the volumes of bilateral amygdala and STAI-T scores in our present study. One possible reason for this negative finding is that the local deformation change with trait anxiety level is too subtle to make the change of entire volume notable. Further investigation is warranted to validate this.

According to a histologically-based probabilistic atlas [35], amygdala can be divided into three subregions: laterobasal group (LB), centromedial group (CM), and superficial group (SF). In our study, the observed significant correlation of surface morphology with trait anxiety were mainly located around LB of right amygdala,which mainly consists of the lateral and central nucleus. It has been demonstrated that the lateral and central nucleus of amygdala mediate reactions and actions elicited by negative stimuli. For instance, Amorapanth and colleagues found fear-arousing stimuli elicited reactive responses through the system composed by lateral nucleus and central nucleus in rats [6]. Another study reported that the basolateral and central nuclei played an important role in processing aversive information when investigating the regulation exerted byγ-Aminobutyric acid mechanisms in rats [48]. In a GAD patient study, Etkin and colleagues observed a significant increase of volume in the right centromedial subregion of amygdala in patients, by using voxel-based morphometry [49]. Moreover, the authors found that the basolateral and centromedial connectivity patterns were significantly less distinct in GAD patients than in healthy control [49]. Compatibly, the results of present study suggested an increased volume of central and lateral volume in right amygdala with high trait anxiety.

It is worth mentioning that a few studies have suggested associations between the volumes or activities of amygdala and depressive level [50], [51]. Given that the effect of anxiety is typically mixed with depressive level, the observed associations among the surface morphology of amygdala and trait anxiety is possibly attributed to the effect of depressive level. To test this, we calculated the correlation between surface morphology and trait anxiety after controlling SDS scores, and still found significant correlation within the surface cluster of right amygdala. This confirmatory result rules out the possibility that the observed association of surface morphology of amygdala with trait anxiety is driven by depressive level.

Nevertheless, a few issues need to be addressed. Firstly, we drew the boundaries of amygdala manually. While the manual extraction is believed to be accurate, it is time-consuming and labor intensive. In future, accurate computational segmentation methods for amygdala need to be developed for automatically extracting the surfaces of amygdala. Secondly, our study included subjects with a relatively small sample size, leading to a less statistical power and therefore possible missing of some significant results on the surface of amygdala. Further studies with larger sample size should be conducted in future. Lastly, gender effects on the subregional shape of amygdala have been observed [22]. The present study however did not take the gender factor into account, because of the small sample size. Further investigation with a larger sample size is warranted to address this issue.
